# Nutritional Risk Assessment and Adverse Events in Patients Undergoing Left Ventricular Assist Device Implantation—A Retrospective Cohort Study Using Hospital Information System

**DOI:** 10.3390/jcm12227181

**Published:** 2023-11-20

**Authors:** Irena Milaniak, Lucyna Tomaszek, Sylwia Wiśniowska-Śmiałek, Izabela Górkiewicz-Kot, Grzegorz Wasilewski, Paulina Kurleto, Michał Kaleta, Dorota Sobczyk, Karol Wierzbicki

**Affiliations:** 1 Faculty of Medicine and Health Sciences, Andrzej Frycz Modrzewski Krakow University, 30-705 Kraków, Poland; pkurleto@afm.edu.pl; 2 Clinical Department of Heart, Vascular Surgery and Transplantology, Cracow Specialist Hospital Named after St. John Paul II, 31-202 Kraków, Poland; swisniowskasmialek@gmail.com (S.W.-Ś.); i.gorkiewicz@szpitaljp2.krakow.pl (I.G.-K.); g.wasilewski@szpitaljp2.krakow.pl (G.W.); m.kaleta@szpitaljp2.krakow.pl (M.K.); d.sobczyk@szpitaljp2.krakow.pl (D.S.); 3 Jagiellonian University Medical College, 31-008 Kraków, Poland; k.wierzbicki@szpitaljp2.krakow.pl

**Keywords:** left ventricular assist device, nutritional risk assessment, adverse events

## Abstract

Clinical or subclinical malnutrition occurs in 30% to 70% of patients with advanced heart failure and increases the risk of postoperative adverse events. The main objective of this study was to assess the nutritional status of patients prior to left ventricular assist device (LVAD) implantation using different methods of malnutrition assessment and to evaluate the relationship between nutritional status and postoperative adverse events. A retrospective cohort study included 120 patients aged 26–74 years referred for LVAD surgery. Preoperative nutritional status (NRS-2002—Nutritional Risk Score 2002, NRI—Nutritional Risk Index, PNI—Prognostic Nutritional Index; TLC—total lymphocyte count) and postoperative adverse events were assessed. Moderate to severe malnutrition was found in 55.8%, 43.3%, 40.0%, and 20% of all patients, respectively, according to the PNI, NRI, TLC, and NRS-2002 scores. Patients with a TLC < 1200 cells/m^3^ had a higher risk of postoperative acute renal failure [hazard ratio (HR): 2.5; 95% confidence interval (95% CI): 1.01–6.3] and death during the observation period [HR = 2.1; 95% CI: 1.2–3.5]. Moderate to severe malnutrition was also associated with a significantly increased risk of in-hospital death [for the NRI score, HR = 4.9 (95% CI: 1.1–22.0); for the PNI score, HR = 5.0 (95% CI: 1.1–22.3)]. In conclusion, moderate to severe malnutrition prior to LVAD implantation has been identified as a risk factor for postoperative acute renal failure and mortality. Assessment of nutritional risk may improve patient selection and early initiation of nutritional support.

## 1. Introduction

Despite tremendous advances in the diagnosis and treatment of cardiovascular disease, heart failure (HF) remains the leading cause of death worldwide [1]. One of the established surgical treatments for advanced HF is long-term mechanical circulatory support [2], mainly left ventricular assist devices (LVADs), which can improve survival and reduce symptoms. The current 1-year survival of patients receiving the latest continuous-flow LVADs is similar to that of heart transplantation, although adverse events negatively impact quality of life (QOL). Thereafter, transplant survival is superior to mechanical support, regardless of indication [2,3].

Malnutrition results from a failure to absorb or use nutrients, leading to altered body composition, impaired physical and mental function, and adversely affecting the outcome of the underlying disease [4]. The progression of heart failure leads to the development of cardiac cachexia, which is a significant predictor of reduced survival in patients with heart failure. Cachexia is defined as a complex metabolic syndrome associated with the underlying disease and is characterized by muscle wasting, which may or may not be accompanied by a loss of fat mass [4,5,6]. Clinical or subclinical malnutrition affects 30% to 70% of patients with advanced heart failure [4]. Based on the research review, severe malnutrition occurs in 7.5% and moderate malnutrition in 57% of all patients. Malnutrition in patients with heart failure increases the risk of mortality by a factor of 2–10 and the risk of hospitalization by a factor of 1.2–1.7 [3,5,6].

On the other hand, cardiac cachexia is estimated to occur in 5–15% of patients with advanced heart failure and is considered a serious complication with a poor prognosis [7]. The pathogenesis of cachexia is unclear, and there appear to be several contributing mechanisms. On the one hand, HF promotes an increase in the catabolic state, and on the other hand, there seems to be an inflammatory and neurohormonal activation that favors the loss of muscle mass and induces anorexia. Gastrointestinal abnormalities also contribute to cardiac cachexia [7].

The primary consequences of malnutrition include a deterioration of the general condition, loss of body weight, decreased muscle mass, muscle strength, and psychomotor performance, impaired immunity, hypochromic anemia, decreased serum protein levels, electrolyte disturbances, dysfunctional intestinal peristalsis and mucosal atrophy, digestive and absorptive disorders, colonization of the small intestine with pathogenic bacteria, decreased renal filtration rate, and metabolic acidosis [7]. The secondary consequences of malnutrition include a higher incidence of infection, impaired wound healing, and a higher incidence of severe illness, leading to prolonged hospital stay and recovery, as well as increased mortality [8].

Most patients with LVADs suffer from moderate to severe malnutrition and deconditioning due to their advanced disease. Malnutrition can increase the risk of device infection, bleeding, and stroke, and thus significantly affect the clinical course of LVADs and the worsening of heart failure [9]. Therefore, the relationship between nutritional status and long-term prognosis after LVAD implantation should be investigated [10].

Several screening tools have been used in patients with HF, but none is the gold standard. Basic methods to assess nutritional status include dedicated scales (Nutritional Risk Screening 2002—NRS-2002, Subjective Global Assessment—SGA, Prognostic Nutritional Index—PNI, Nutritional Risk Index—NRI), dietary history, and anthropometric examinations (current body weight, unintentional weight loss, body mass index—BMI, arm circumference, triceps skinfold thickness, handgrip strength, bioelectrical impedance analysis) [11]. Biochemical tests provide an objective measure of malnutrition. Proteins, such as albumin, transferrin, prealbumin, and immunologic tests, such as the total lymphocyte count, are crucial in assessing nutritional status [12,13].

In Poland, despite the mandatory screening of nutritional status in hospitalized patients [14], there are no studies on the problem of malnutrition in patients with heart failure and mechanical circulatory support. Hospitalization rates for HF in Poland are among the highest in Europe at 547:100,000 population [15]. In view of these data, it is worth recalling that the severity of heart failure and malnutrition affect mortality and the length of hospital stay. Therefore, the assessment of nutritional status and early nutritional intervention play an important role in patient prognosis and the reduction in HF-related costs.

The aim of this study was to evaluate (1) the risk associated with nutritional status in patients eligible for mechanical circulatory support implantation in the preoperative period, and (2) the prognostic significance of malnutrition indicators in the postoperative period. The former will be achieved by applying different methods of malnutrition risk assessment.

## 2. Materials and Methods

### 2.1. Study Design, Setting, and Ethical Considerations

This was a retrospective cohort study. We analyzed 120 electronic health records of patients aged 26–74 years who were referred for LVAD implantation between October 2015 and December 2022. The study followed the guidelines of the RECORD statement (the reporting of studies conducted using observational routinely collected health data) [16]. This study was conducted at the Clinical Department of Cardiovascular Surgery and Transplantology, Krakow, St. John Paul II Hospital, Poland (approved by the Bioethics Committee; Opinion No. 1072.6120.253.2021).

### 2.2. Participants

All patients, both sexes, aged at least 18 years, with a diagnosis of HF and referred for LVAD therapy by the Heart Failure Team were included. Duplicates and those with incomplete data were excluded.

### 2.3. Variables

Demographics included age and sex. Other data collected before and after LVAD implantation are shown in Table 1. Indications for LVAD implantation were according to the European Society of Cardiology (ESC) guidelines [3]. INTERMACS profiles provide prognostic information for patients with advanced heart failure on mechanical support [3]. In this study, right and left ventricular function and tricuspid annular plane systolic excursion (TAPSE) were assessed (TAPSE > 1.9 cm was normal) [17]. Left ventricular function was described according to the classification of heart failure by the left ventricular ejection fraction (LVEF), where LVEF ≤ 30% was a cut-off value for advanced heart failure [3]. A hospital admission infection risk assessment scale was used to determine the risk of infection prior to LVAD surgery. The total score ranges from 0 to 26. The higher the score, the higher the risk of infection.

Nutritional status was assessed using BMI according to the World Health Organization (WHO) criteria [18].

The NRS-2002 includes standard screening parameters such as body mass index (BMI), patient age, weight loss, dietary intake, and severity of the underlying disease. Impaired nutritional status and disease severity are scored on a scale of 0 to 3. Patients aged 70 years or older receive an additional point. The age-adjusted NRS-2002 total score ranges from 0 to 7. A score equal to or above three indicates a high nutritional risk, while a score below three places the patient in the low nutritional risk group. An NRS-2002 calculator is available (https://www.mdcalc.com/calc/4012/nutrition-risk-screening-2002-nrs-2002, accessed on 30 September 2023) [19,20,21].

The NRI is an index based on ideal body weight that aims to represent body weight and serum albumin levels. The NRI was calculated as (1.5 × serum albumin [g/L] + 41.7 × [current body weight]). A patient with an NRI of >100 is considered in the no-risk group, 97.5–100—mild risk, 83.5–97.5—moderate risk, and <83.5—severe risk. An NRI calculator is available (https://www.mdcalc.com/calc/4012/nutrition-risk-screening-2002-nrs-2002, accessed on 30 September 2023) [22].

The PNI was calculated as (10 × serum albumin (g/dL) + 0.005 × total lymphocytes (1000/μL)). A score < 30 indicates severe malnutrition, PNI > 38 is normal, and 35–38 indicates a moderate risk of malnutrition [23].

Total lymphocyte count (TLC) was calculated as % lymphocytes × lymphocyte value/100. The cut-off points used to classify nutritional status are >1500 cells/m^3^ (normal), 1200 to 1499 cells/m^3^ (mild risk), 800 to 1199 cells/m^3^ (moderate risk), and <800 cells/m^3^ (severe risk) [24].

The time to an adverse event includes the time from LVAD implantation to the occurrence of an adverse event during hospitalization (bleeding, infection, respiratory and acute renal failure, death) or during the observation period, i.e., until 31 December 2022 (stroke, death). Any patient who survived was censored (time to adverse event information was not available because the event did not occur before the end of the study). Respiratory failure was defined as pulmonary insufficiency requiring intubation and mechanical ventilation for 96 h or more during the postoperative period or because of inadequate noninvasive ventilation. Acute renal failure was estimated by an estimated glomerular filtration rate [eGFR] < 30 mL/min/1.73 m^2^, calculated from the Modification Of Diet In Renal Disease [MDRD] or Chronic Kidney Disease Epidemiology Collaboration formulas [25], and a new need for dialysis or an increase in serum creatinine to three times its baseline value. Bleeding was defined as any episode of internal or external bleeding resulting in death, reoperation, hospitalization, or red blood cell transfusion. Infection was defined as any infection proven by microbiological isolation requiring treatment with intravenous antibiotics during the postoperative period.

### 2.4. Outcomes

The primary outcome was nutritional assessment prior to LVAD implantation. The secondary outcome was adverse events after LVAD implantation, including bleeding, infection, respiratory and acute renal failure, stroke, and death.

### 2.5. Statistical Analysis

Continuous data are presented as medians (25th–75th percentiles), whereas categorical variables are presented as numbers and percentages. Intergroup differences for continuous and categorical variables were assessed using Mann–Whitney or chi-squared tests (or Fisher’s exact test), respectively. Correlations between numerical parameter values were determined using Spearman’s (R) rank correlation coefficient. The distribution of variables was tested using the Shapiro–Wilk test. Kaplan–Meier curves were plotted, and log-rank tests were used to compare progression-free survival for secondary outcomes (adverse events) according to nutritional risk. Cox proportional hazards regression analysis was used to examine the relationship between dietary risk and secondary outcomes. Results are presented as hazard ratios (HR) with 95% CIs. All statistical analyses were performed with STATISTICA v.13 (TIBCO Software Inc., Kraków, Poland, 2017). A *p*-value < 0.05 was considered statistically significant.

## 3. Results

### 3.1. Baseline Characteristic of Patients before LVAD Implantation

In total, 120 patients (114 men, 95%), median age 63 years, underwent LVAD implantation. Herein, 55.8% of patients received the HeartMate III device, and the Heart Ware system was implanted in 44.2%. The length of hospital stay ranged from 3 to 90 days. The most common indication for surgery was bridging to transplantation (66.7%). The majority of pre-implant patients were in INTERMACS profile 3 (56.7%). The median TAPSE was 1.6 cm; 15.8% of patients had a normal TAPSE. The median LVEF was 15%. The most common comorbidity was diabetes (about 50%). In the preoperative period, nutritional supplements were ordered for five patients. None of the patients were treated with tube feeding or parenteral nutrition. The preoperative patient characteristics are shown in Table 2.

### 3.2. Nutritional Assessment

Only two patients had a BMI below 18.5, indicating underweight, while the remaining patients had BMIs within the normal range (n = 29, 24.2%) or overweight (n = 89, 74.2%). According to the NRS-2002 scale, one in five patients (20.0%) was at a high nutritional risk prior to LVAD implantation. According to the PNI, NRI, and TLC scores, the prevalence of a moderate to severe malnutrition risk was observed in 55.8%, 43.3%, and 40.0% of all patients, respectively.

### 3.3. Postoperative Outcomes

Bleeding was the second most common adverse event (39.2%) after infection (40.0%). Respiratory failure and acute renal failure occurred in 21.2% and 19.2% of all patients, respectively. The in-hospital mortality rate was 14.2%. During a median follow-up of 696 days, 52.5% of all patients died.

The median length of hospital stay after LVAD implantation was 30 days (range 3–120 days). Patients with bleeding (median 35 vs. 28; Z = −2.53; *p* = 0.01), infection (median 32 vs. 28; Z = −2.08; *p* = 0.04), respiratory failure (median 36 vs. 30; Z = 2.19; *p* = 0.03), and a higher risk of NRS-2002 (median 37 vs. 28; Z = −3.07; *p* = 0.002) required longer hospital stays. There was no significant correlation between the length of hospital stay before and after LVAD implantation (R = 0.14; t = 1.59; *p* = 0.11).

### 3.4. The Prognostic Significance of Malnutrition Indicators in Predicting Postoperative Adverse Events

Analysis of Kaplan–Meier curves showed that some indicators of nutritional status were predictors of secondary outcomes: acute renal failure (TLC), in-hospital mortality (PNI, NRI), and all-cause mortality (TLC). The predictors of postoperative adverse events after LVAD are summarized in Table 3.

### 3.5. Acute Renal Failure

To evaluate the predictive value of TLC, we categorized patients into two malnutrition risk groups: normal to mild (TLC ≥ 1200 cells/m^3^) vs. moderate to severe (TLC < 1200 cells/m^3^) (Table 2). The incidence of acute renal failure was 9.7% (n = 7) in the normal to mild group and 33.3% (n = 16) in the moderate to severe group (X^2^ = 10.36; *p* = 0.001).

**Figure 1:**
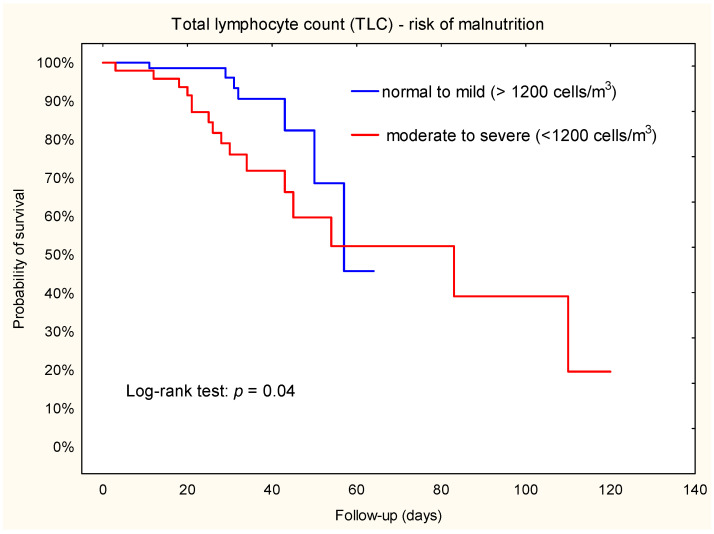
Kaplan–Meier plot. Association between TLC and acute renal failure during hospital stay after left ventricular assist device implantation.

The results of Kaplan–Meier survival analysis showed that patients with a TLC < 1200 cells/m^3^ had a higher risk of renal failure compared to TLC ≥ 1200 cells/m^3^ (Figure 1). The Cox proportional hazard model further demonstrated that patients in the TLC < 1200 cells/m^3^ group had a 2.5-fold increased risk of renal outcome compared to patients in the higher TLC group (HR = 2.5; 95% CI: 1.01–6.3).

### 3.6. Mortality during the Hospital Stay

During follow-up in the group stratified by NRI, more patients died in the moderate to severe group than in the normal to mild group (n = 15; 22.1% vs. n = 2; 3.8%; X^2^ = 6.61; *p* = 0.007). Similar results were observed in groups stratified by PNI: 15 patients died in the moderate to severe group and 2 in the normal group (X^2^ = 6.97; *p* = 0.003).

**Figure 2:**
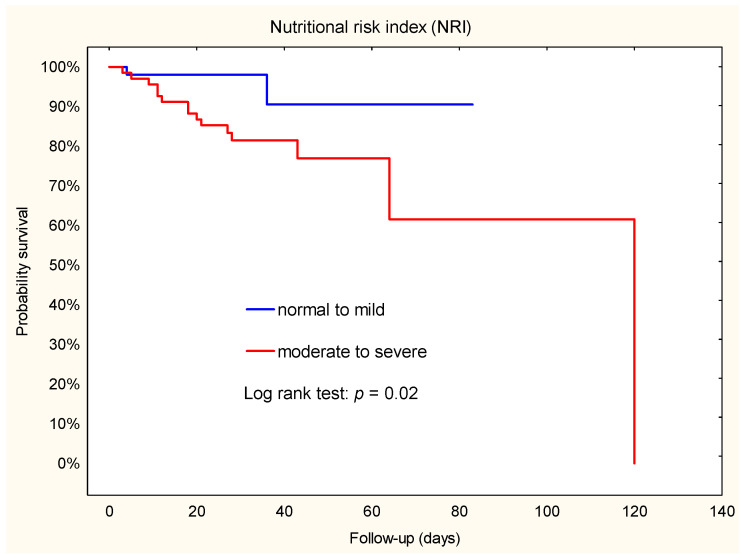
Kaplan–Meier plot. Association between NRI and death during hospital stay after implantation of a left ventricular assist device.

**Figure 3:**
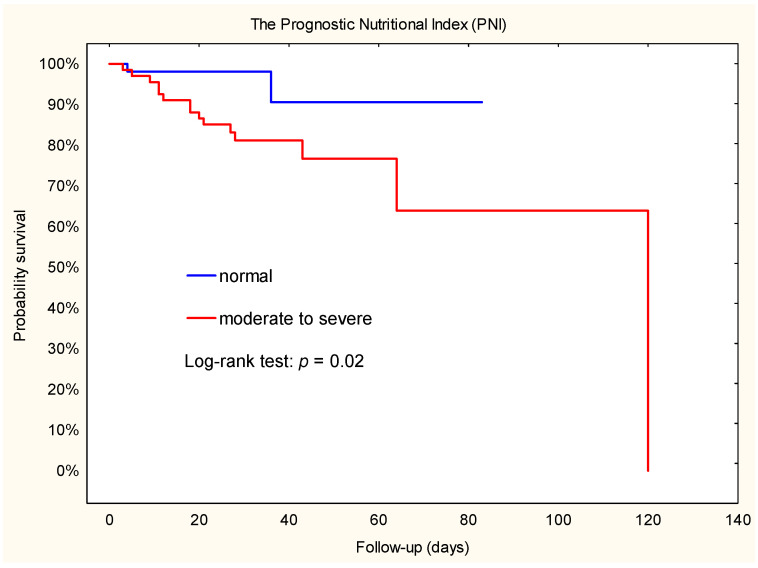
Kaplan–Meier plot. Association between PNI and death during hospital stay after implantation of a left ventricular assist device.

Kaplan–Meier survival estimates showed that patients with a moderate to severe risk of malnutrition had a higher risk of death compared to those with a normal to mild risk of NRI or normal risk of PNI (Figure 2 and Figure 3). In addition, Cox regression analysis showed that patients with a moderate to severe risk of malnutrition had a 5-fold-increased risk of death. Furthermore, the hazard ratio for NRI was 4.9 (95% CI: 1.1–22.0), while the hazard ratio for PNI was 5.0 (95% CI: 1.1–22.3).

### 3.7. Mortality throughout the Observation Period

**Figure 4:**
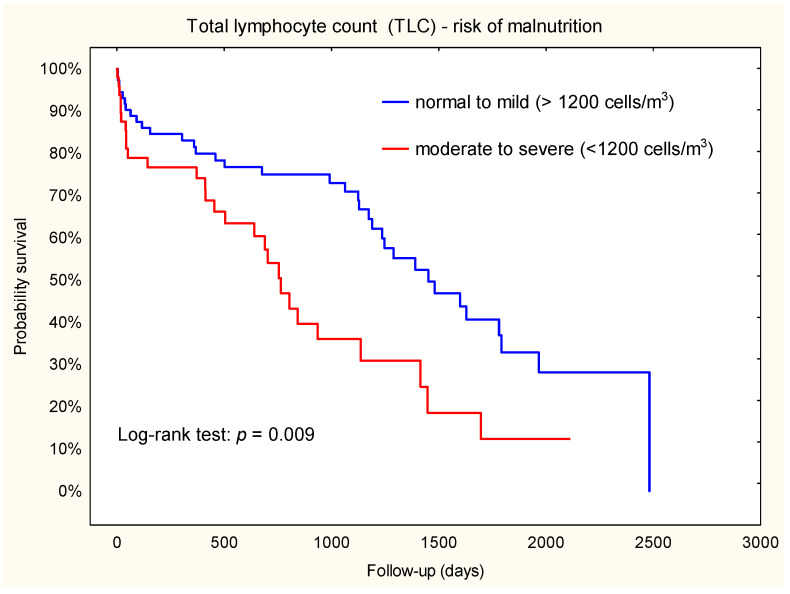
Kaplan–Meier plot. Association between TLC and death throughout the observation period.

During a median follow-up of 696 days, 28 patients (58.3%) died in the TLC < 1200 cells/m^3^ group and 35 patients (48.6%) died in the TLC ≥ 1200 cells/m^3^ group. Kaplan–Meier survival analysis was used to evaluate the relationship between TLC subsets and death (Figure 4). Our results showed that patients with a TLC < 1200 cells/m^3^ had a higher risk of death compared to patients with a TLC ≥ 1200 cells/m^3^. This finding confirmed the results of the Cox regression analyses (HR = 2.1; 95% CI: 1.2–3.5).

## 4. Discussion

This study retrospectively evaluated the impact of LVAD nutritional status in the early postoperative period using data from the hospital information system. This study showed that the incidence of malnutrition in patients before LVAD implantation ranged from 20 to 55.8%, depending on the malnutrition screening tools used. As a result, moderate to severe malnutrition before LVAD was recognized as a risk factor for postoperative acute renal failure and mortality.

In our study, only two patients were underweight according to BMI. In contrast, the NRS-2002 identified one in five patients as being at high nutritional risk before LVAD implantation. We found that anthropometric assessment had some limitations, i.e., fluid retention without direct correlation to nutritional status. Felpel et al. came to similar conclusions [26]. In our study, the risk of moderate to severe malnutrition according to PNI, NRI, and TLC was assessed in 55.8%, 43.3%, and 40.0% of the patients, respectively. Felpel et al. found similar results for NRI scores [26]. Uribarri et al. demonstrated that the prevalence of severe, moderate, and mild nutritional risk was 5.4%, 21.5%, and 9.3%, respectively, among all LVAD candidates [27], while Gülleroğlu et al. found a higher mean NRI score (99.6 ± 10.2) in LVAD patients (93.5 ± 8.8). An NRI score of 32 (53.3%) indicates the risk of malnutrition. Gülleroğlu et al. classified the risk of malnutrition as mild, moderate, and severe in 6 (10%), 25 (41.6%), and 1 (1.7%) cases, respectively [28]. Yost et al. showed a mean PNI for LVAD candidates of 30.1 ± 4.6, indicating widespread malnutrition [29]; we achieved higher results (35.9 ± 5.8).

Total lymphocyte count (TLC) and serum albumin are reliable nutritional indices [30]. In our study, the prevalence of a moderate-severe risk of malnutrition according to TLC was observed in 40% of patients. In a study evaluating TLC and albumin in surgical patients, malnutrition was higher than in our study (73.9% by TLC). In contrast, Tojek et al. reported in a retrospective analysis of 54,976 patients that a TLC < 0.8 G/L occurred in 15% of patients [31].

Complications of LVAD therapy include bleeding, infection, pump thrombosis, right ventricular failure, device malfunction, and stroke [32]. The following postoperative adverse events were observed in our study: infection, bleeding, respiratory failure, and acute renal failure. Stroke occurred in 14.2% of patients during the entire observation period, and the mortality rate during the first hospitalization was 14.2%. These results correlate with the data of Yuzefpolskaya et al. in the STS INTERMACS 2022 Annual Report [33] and the report of Jezovnik et al. [34]. In our study, malnutrition rates were associated with adverse events; patients with a TLC < 1200 cells/m^3^ had a 2.5-fold risk of acute renal failure. Schrot et al. found in their meta-analysis that lymphopenia was associated with acute renal failure [35].

Mechanical circulatory support can improve survival and reduce symptoms in patients with advanced HF, with 1-year survival rates of up to 83% and 5-year survival rates of up to 51.9% for LVADs. To achieve high survival rates, candidates should be carefully selected and well prepared [33]. In our study, 52.5% of all patients died during a median follow-up of 696 days. Here, heart failure was often associated with weight loss, culminating in cardiac cachexia in advanced stages [2]. Our study showed that only NRI and PNI had predictive value for in-hospital mortality.

Our results are consistent with those of Yost et al., who found that a PNI of less than 30 was associated with a 12.2% reduction in postoperative survival [29]. Uribarri et al. showed that a normal preoperative NRI was an independent predictor of a lower risk of death from any cause [27].

Our results showed that lymphopenia with a TLC < 1200 cells/mm^3^ is a good predictor of mortality risk. Majmundar et al. observed that a TLC less than or equal to 1500 cells/mm^3^ in HF patients correlated with a high risk of mortality [36]. Charach et al. also showed that TLC was inversely associated with predicted mortality, with low lymphocyte counts (<1600 median) decreasing the 8-year survival compared to those 1600 (58% vs. 72%) [37]. The results of Stawiarski et al. confirmed a significantly higher all-cause mortality in the lymphopenia group [38]. In the overall assessment of the patient, it must be considered that the albumin level, pre-albumin level, white blood cell count, and lymphocyte count used in the nutritional risk scores are also biomarkers of inflammation [26]. Pre-albumin has a shorter half-life than albumin, is not affected by hydration status or renal function, and is a more sensitive protein indicator of nutritional status. However, the single-center study showed that low preoperative pre-albumin levels are associated with increased mortality after LVAD implantation; future studies should evaluate whether preoperative interventions to optimize nutritional status can improve outcomes in patients with poor nutrition. Also, this is not a routine test in patient assessment [28].

Malnutrition and frailty are recognized factors for poor postoperative outcomes in the LVAD population. In our study, the majority of patients were INTERMACS 2 (progressive decline) and 3 (stable but inotrope dependent) and were scheduled for elective surgery. In patients with heart disease, preoperative oral nutritional supplements administered in the setting of elective cardiac surgery and patients with heart failure show feasibility, safety, and good patient compliance [39]. Multimodal prehabilitation has also emerged in recent years as an innovative intervention that focuses on optimizing the patient’s physical function, nutritional and psychological status, and optimizing the management of existing comorbidities [40].

### Limitations

The main limitations are retrospective and single-center patterns; therefore, the results may not be generalizable. The most recent available blood tests before cf-LVAD implantation were used, but we cannot exclude the possibility of their variation before the procedure.

## 5. Conclusions

In our study, malnutrition in LVAD candidates ranged from 20% to 55.8%, depending on the method used to assess malnutrition risk. We found that the presence of malnutrition effectively predicted postoperative adverse events according to preoperative NRI, PNI, and TLC scores. Malnourished patients are at an increased risk for postoperative complications and death. The assessment of nutritional risk could improve patient selection and allow early nutritional intervention to improve survival. Our results highlight the need for continued investigation of the potential clinical benefit of nutritional intervention in LVAD candidates. However, the NRS-2002 scale is recommended by many societies for nutritional screening in heart failure patients, and our study suggests that it is not a good predictor of mortality after LVAD because of significant fluid retention.

Implications for practice. Nutritional risk assessment may improve patient selection and allow early nutritional intervention to improve survival. Our results highlight the need to further investigate the potential clinical benefit of nutritional intervention in LVAD candidates. Therefore, all LVAD patients should be considered high risk and receive appropriate nutritional interventions.

## Figures and Tables

**Table 1:** Data collected before and after implantation of a left ventricular assist device (LVAD).

Preoperative Variables			Postoperative Outcomes
Clinical Data	Laboratory Test Data	Nutritional Assessment	Adverse Events
LVAD type Diagnosis Comorbidity LVAD indications INTERMACS TAPSE LVEF Inotropic support Risk of infection Length of hospital stay (days)	Alanine aminotransferase Aspartate aminotransferase Bilirubin, total Blood urea nitrogen Brain natriuretic peptide Cholesterol, total eGFR Hemoglobin INR Leukocytes, total Lymphocytes Protein, total Serum albumin Serum creatinine Serum uric acid Serum sodium	BMI NRS-2002 NRI PNI TLC	Respiratory failure Acute renal failure Bleeding Infection Stroke Death

BMI—body mass index; eGFR—estimated glomerular filtration rate; INR—International Normalized Ratio, INTERMACS—Interagency Registry for Mechanically Assisted Circulatory Support; LVEF—left ventricular ejection fraction; NRI—Nutritional Risk Index; NRS-2002—Nutritional Risk Score 2002; PNI—Prognostic Nutritional Index; TAPSE—tricuspid annular plane systolic excursion; TLC—total lymphocyte count.

**Table 2:** Baseline characteristics of patients before and after implantation of a continuous-flow left ventricular assist device (LVAD).

Variables	
Preoperative Variables	
Age (years)	63 [55; 67]
Sex, men	114 (95.0)
Device type:	
• Heart Ware	53 (44.2)
• HeartMate III	67 (55.8)
Aetiology of heart failure	
• Dilated cardiomyopathy	70 (58.3)
• Ischemic cardiomyopathy	50 (41.7)
Comorbidities:	
• Diabetes	41 (34.2)
• Chronic kidney disease	16 (13.3)
• Diabetes + chronic kidney disease	15 (12.5)
• Other	8 (6.7)
INTERMACS patient profile:	
• 1	11 (9.2)
• 2	37 (30.8)
• 3	68 (56.7)
• 4	4 (3.3)
LVAD treatment indications:	
• Bridge to transplantation	80 (66.7)
• Bridge to decision-making	32 (26.7)
• Bridge to candidacy	5 (4.2)
• Destination therapy	3 (2.5)
Inotropic support	102 (85)
Risk of infection	3 [1; 4]
Tricuspid annular plane systolic excursion (TAPSE, cm)	1.6 [1.5; 1.8]
Left ventricular ejection fraction (LVEF; %)	15 [10; 15]
Length of hospital stay (days)	16 [9; 23]
Laboratory data:	
• Alanine aminotransferase (U/L)	24 [15; 37]
• Aspartate aminotransferase (U/L)	22 [17; 30]
• Bilirubin, total (μmol/L)	16.2 [10.8; 25.7]
• Blood urea nitrogen (mmol/L)	7.3 [5.4; 10.3]
• Brain natriuretic peptide (pg/mL)	3592 [2139; 6938]
• Cholesterol, total (mmol/L)	3.5 [3.0; 4.3]
• eGFR ml/min/1.73m^2^	69 [57; 86]
• INR	1.1 [1.0; 1.2]
• Hemoglobin (g/dL)	12.9 [11.9; 14.3]
• Leukocytes, total (1000/μL)	7.6 [6.2; 9.5]
• Protein, total (g/L)	57.6 [54.0; 61.5]
• Serum albumin level (g/L)	35 [30; 38]
• Serum creatinine (μmol/L)	106 [80; 124]
• Serum uric acid (μmol/L)	386 (308; 456)
• Serum sodium (mmol/L)	138 [136; 141]
Nutritional assessment—the risk of malnutrition:	
• Body mass index (BMI, kg/m^2^)	27.0 [24.8; 30.8]
• Nutritional Risk Score 2002 (NRS-2002)	0.0 [0.0; 1.5]
Low	96 (80.0)
High	24 (20.0)
• Nutritional Risk Index (NRI)	94.2 [87.3; 99.1]
Normal to mild	52 (43.3)
Moderate to severe	68 (56.7)
• Prognostic Nutritional Index (PNI)	37.0 [32.3; 40.1]
Normal	53 (44.2)
Moderate to severe	67 (55.8)
• Total lymphocyte count (TLC, cells/m^3^)	1440 [862; 2031]
Normal to mild	72 (60.0)
Moderate to severe	48 (40.0)
Postoperative Outcomes	
• Infection	48 (40)
• Bleeding	47 (39.2)
• Respiratory failure	26 (21.2)
• Acute renal failure	23 (19.2)
• Stroke	17 (14.2)
• Length of hospital stay (days)	30 [24; 37]
• Length of observation period (days)	696 [225; 1263]
• Mortality during hospital stay	17 (14.2)
• Mortality throughout the observation period	63 (52.5)

Continuous variables are presented as medians [upper and lower quartiles] and categorical variables as absolute numbers (percentages).

**Table 3:** The prognostic significance of malnutrition indicators in predicting postoperative adverse events after implantation of a continuous-flow left ventricular assist device.

Predictors	Reference Category	Postoperative Adverse Events	Risk of Postoperative Adverse Events
TLC < 1200 cells/m^3^	TLC ≥ 1200 cells/m^3^	Acute renal failure	HR = 2.5; 95% CI: 1.01–6.3
		Mortality throughout the observation period	HR = 2.1; 95% CI: 1.2–3.5
NRI moderate to severe	NRI normal to mild	Mortality during the hospital stay	HR = 4.9; 95% CI: 1.1–22.0
PNI moderate to severe	PNI normal	Mortality during the hospital stay	HR = 5.0; 95% CI: 1.1–22.3

TLC—total lymphocyte count; NRI—Nutritional Risk Index; PNI—Prognostic Nutritional Index; HR—hazard ratio; CI—confidence interval.

## Data Availability

Data are contained within the article.
